# Efficacy of cognitive rehabilitation on psychosocial functioning in Borderline Personality Disorder: a randomized controlled trial

**DOI:** 10.1186/s12888-015-0640-5

**Published:** 2015-10-21

**Authors:** Juan C. Pascual, Nerea Palomares, Ángela Ibáñez, Maria J. Portella, Rocío Arza, Raquel Reyes, Albert Feliu-Soler, Marina Díaz-Marsá, Jerónimo Saiz-Ruiz, Joaquim Soler, Jose L. Carrasco

**Affiliations:** Department of Psychiatry, Hospital de la Santa Creu i Sant Pau, Barcelona, Spain; Centro de Investigación Biomédica en Red de Salud Mental (CIBERSAM), Institut d’Investigació Biomèdica - Sant Pau (IIB-Sant Pau), Universitat Autònoma de Barcelona, Av. Sant Antoni Ma Claret 167, 08025 Barcelona, Spain; Instituto de Investigación Sanitaria del Hospital Clínico San Carlos, Madrid, Spain; Centro de Investigación Biomédica en Red de Salud Mental (CIBERSAM), Departamento de Psiquiatría, Universidad Complutense, Madrid, Spain; Department of Psychiatry, Universitary Hospital Ramón y Cajal, CIBERSAM, IRYCIS, Madrid, Spain; Department of Psychiatry, Universidad de Alcalá, Madrid, Spain; FIBio-HRC, Universitary Hospital Ramón y Cajal, Madrid, Spain

**Keywords:** Borderline personality disorder, Cognitive rehabilitation, Psychoeducation, Psychosocial functioning

## Abstract

**Background:**

Follow-up studies revealed that subjects with borderline personality disorder (BPD) present high rates of clinical remission, although psychosocial functioning often remains impaired. The aim of this study is to evaluate the efficacy of a cognitive rehabilitation intervention versus a psychoeducational program on psychosocial functioning in subjects with BPD.

**Methods:**

A multicenter, randomized, and positive-controlled clinical trial was conducted. Seventy outpatients with BPD were randomized to cognitive rehabilitation or psychoeducational group interventions. Participants were evaluated after completion of the intervention period (16 weeks) and after the follow-up period (6 months). Psychosocial functioning, clinical and neuropsychological outcomes were evaluated.

**Results:**

No main effects of group or group x time were observed on functionality but a significant effect of time was found. *Post-hoc* analyses showed that only cognitive rehabilitation increased psychosocial functioning significantly at endpoint. Psychoeducation showed a significant enhancement of depressive symptoms.

**Conclusions:**

Cognitive rehabilitation and psychoeducational interventions appeared to show good efficacy in improving disabilities in daily life in subjects with BPD. These interventions are easily implemented in mental health settings and have the advantage of improving general functioning and clinical symptoms.

**Trial registration:**

Clinicaltrials.gov:NCT02033044. Registered 9 January 2014

**Electronic supplementary material:**

The online version of this article (doi:10.1186/s12888-015-0640-5) contains supplementary material, which is available to authorized users.

## Background

Borderline Personality Disorder (BPD) is a common and severe disorder that has long been considered a chronic and untreatable disorder for many clinicians [1, 2]. Nevertheless, recent long follow-up studies offer a more optimistic scenario indicating high rates of clinical remission [2–4]. However, remission does not appear to be equivalent to full recovery as symptoms improvement is not necessarily associated with amelioration of psychosocial dysfunctioning. These same studies point out that the challenge for the next generation of therapies is to redirect the focus onto improving functional outcomes as well as clinical symptoms [2–4]. Most psychotherapies such as Dialectical Behaviour Therapy (DBT) or Mentalization Based Therapy have proven their efficacy to treat emotional dysregulation, impulsivity and interpersonal difficulties, but not cognitive deficits or psychosocial functioning [1].

It is often accepted that psychosocial dysfunction is partly caused by cognitive deficits that remained impaired after clinical remission in other mental disorders such as schizophrenia and bipolar disorder [5, 6], but there are few studies addressing this issue in BPD. Some recent studies have established that neuropsychological dysfunction of BPD might be affecting domains such as attention, cognitive flexibility, memory, planning, processing speed and visuo-spatial skills [7–10]. Accordingly, recent neuroimaging studies in BPD have reported structural and functional abnormalities in many brain areas supporting the assumption of a dysfunctional frontolimbic network in subjects with BPD [1, 11–15]. Such abnormalities are compatible with the cognitive impairment observed in BPD patients [7–10]. Nevertheless, some authors suggest that these neurocognitive dysfunctions in BPD may owe more to the impact of transient mood states or emotional distress than to underlying primary cognitive deficits per se [16, 17].

Cognitive rehabilitation strategies have usually been applied in schizophrenia [18], and, more recently, their efficacy has been demonstrated in affective disorders [19, 20]. To our knowledge, there are no consistent data on the application of neuropsychological remediation in patients with BPD, apart from preliminary communications of positive findings in case series [21, 22]. The aim of the present randomized and controlled study is to evaluate the efficacy of a cognitive rehabilitation group therapy as compared to a psychoeducational group intervention in subjects with BPD on psychosocial functioning.

## Method

### Participants

A total of 70 outpatients with BPD were included from September 2011 to July 2013, 30 from the *Hospital Clinico San Carlos*, 20 from the *Hospital Santa Creu i Sant Pau*, and 20 from the *Hospital Ramón y Cajal*. Inclusion criteria were the following: 1) Outpatients aged 18 to 45 years; 2) Diagnoses of BPD according to DSM-IV-TR [23] criteria and evaluated by two semi-structured diagnostic interviews -Structured Clinical Interview for DSM-IV Axis II Disorders (SCID-II) [24] and the Revised Diagnostic Interview for Borderlines (DIB-R) [25]-to guarantee a correct diagnosis; 3) Clinical severity measured with Clinical Global Impression for BPD (CGI-BPD) [26] higher than 4; and 4) Functional impairment measured with a Global Assessment Functioning (GAF) [23] lower than 65.

Exclusion criteria were the following: 1) Severe physical conditions, such as organic brain syndrome or neurological disease that could affect neuropsychological performance; 2) Intelligence Quotient IQ < 85; 3) Major Depression Disorder (MDD) or substance misuse within the last 6 months evaluated with DSM-IV criteria and SCID-I specific sections; 4) DSM-IV criteria for Schizophrenia, severe psychotic disorder or bipolar disorder evaluated by SCID-I specific sections; 5) Previous participation in any psychoeducation or cognitive rehabilitation intervention.

### Study design and procedure

Study design was a multicenter, randomized, rater-blind clinical trial (see Additional file 1: Figure S1)*.* There were two-parallel arms (1:1) to evaluate functional, clinical and cognitive efficacy of a specific cognitive rehabilitation group intervention (CR) compared with a psychoeducational group intervention (PE) in subjects with BPD. To ensure the reliability among centers regarding the evaluation and the treatment fidelity, two meetings were organized before the start of the study to train therapists.

Clinical and neuropsychological evaluations before interventions were administered on different days since they lasted in general more than one hour each and the effect of fatigue or boredom might have affected the results. Experienced psychiatrists and psychologists performed clinical interviews over 3 months to ensure the follow-up of all participants prospectively. The following sociodemographic and clinical variables were collected: age at recruitment, gender, education level, occupational status, and pharmacological treatment. All participants were randomized to receive CR or PE in a 1:1 ratio stratified by centre, age, and education level. Generation of random allocation sequence was done with the Research Randomizer (www.randomizer.org). The present study was powered to test hypotheses about potential between-treatment differences on the primary outcome. With a sample size of 70 and an expected attrition from assessments of 30 %, the study had power of 65 % and a level of significance of 5 % to detect a moderate effect (d = .6).

Participants were evaluated at baseline, after the intervention (16-week period), and after the follow-up period (six months after the intervention). During the whole study period, subjects did not receive any other individual or group psychotherapy. All patients continued pharmacological treatment if it had been initiated prior to inclusion. Type and doses of medication could not be modified at any time during the whole study period. Both interventions were applied in a group format and were conducted by two psychologists with experience in managing patients with BPD. Subjects were instructed not to disclose any information about the intervention to maintain blind conditions. Adverse events such as severe self-harm, suicide attempt, hospitalization and death during the trial were collected.

### Ethics

The study was approved by the Ethics Committee of the Hospital Clínico San Carlos and carried out in accordance with the ethical principles of Declaration of Helsinki. All subjects received extensive information about the study and provided written informed consent before they were enrolled in the study. This study was registered at clinicaltrials.gov (NCT02033044).

### Interventions

#### Cognitive Rehabilitation (CR)

CR consisted of group sessions (5 individuals per group) of 120 min, twice a week during a total period of 16 weeks (32 sessions). The exercises addressed neurocognitive issues related to sustained attention, processing speed, memory and executive functioning. The whole program aimed at getting new strategies to improve functional adaptation, thus tasks were carried out in the clinical setting and at home. Some homework tasks were based on their daily life difficulties and problems. The main objective of this program was the generalization of rehabilitated cognitive functions to daily life activities. Most of the techniques were based on a previous program for bipolar disorder [20].

#### Psychoeducation (PE)

The psychoeducational intervention consisted of 16 weekly group sessions of 5 individuals of 120 min each (16 sessions). This therapy aimed at improving awareness of illness, interpersonal abilities, family balance, therapeutical adherence, emotional management in frustrating situations, problem solving, and lifestyle regularity. During this intervention, no homework tasks were required. This intervention was based on the first step of the Systems Training for Emotional Predictability and Problem Solving (STEPPS) program: “Awareness of Borderline Personality Disorder” [27]. Any other STEPPS’s contents were included in this psychoeducation intervention. In order to provide a more rigorous comparison condition and for controlling nonspecific effects of the CR (e.g., attending in a regular basis to a group therapy), PE was elected instead of other common comparisons such as treatment as usual (TAU) or waiting list.

### Instruments

#### Diagnostic interviews and severity assessment

- *Structured Clinical Interview for DSM-IV Axis II Personality Disorders* (SCID-II) [24]. A semi-structured interview to assess personality disorders according to DSM-IV criteria. The Spanish version has good discrimination between Axis II personality disorders, as well as good reliability between raters as indicated by an overall Kappa of 0.85.
- *Revised Diagnostic Interview for Borderlines* (DIB-R) [25]. The DIB-R is a semi-structured interview that brings the diagnosis of BPD within the last two years. Scores range from 0 (no BPD severity) to 10 (high BPD severity). The Spanish version has shown good psychometric properties regarding internal consistency (Cronbach’s alpha: 0.89), sensitivity (0.81) and specificity (0.94). The interviewers were experienced psychologists and presented a high inter-rater reliability (within-class correlation: 0.94). The inter-test-reliability of DIB-R and SCID-II was moderate (Kappa: 0.59).
- *Clinical Global Impression Scale for BPD (CGI-BPD)* [26]. This clinician-rated scale assesses global severity for BPD and symptomatic dimensions using a 7-point Likert scale, which ranges from 1 (absence of illness) to 7 (high severity of illness).
- *Global Assessment Functioning (GAF)* [23], from the Diagnostic and Statistical Manual of Mental Disorders, Fourth Edition Text Revision (DSM-IV-TR). This scale allows the evaluation of the global functioning of the patient and ranges from 1 to 100.

### Primary outcome: psychosocial functioning

- *Functioning Assessment Scale Test (FAST)* [28]. This was the primary outcome measure. It is a 24-item clinician-rated scale that measures the level of functioning of patients in daily life situations in the past two weeks. It assesses six functional domains: autonomy, occupational functioning, cognitive functioning, financial issues, interpersonal relationships, and leisure time. Scores range from 0 (none) to 3 (higher functional impairment). This instrument has shown good psychometric properties and sensitivity to change.

### Secondary outcomes: clinical and neuropsychological effects

#### Clinical assessment

- *Borderline Symptom List – 23 (BSL-23)* [29]. A 23-item self-rating instrument used to assess the typical symptomatology and severity of BPD. It is rated by using a 5-point scale from 0 (“not at all”) to 4 (“very strong”). This instrument has shown good psychometric properties.
- *Hamilton Anxiety Rating Scale (HARS)* [30]. It is a clinician-rated scale to evaluate current anxious symptoms.
- *Montgomery-Asberg Depression Rating Scale (MADRS)* [31]. This is a self-rated questionnaire for the evaluation of depression severity.
- *Barrat Impulsivity Scale (BIS)* [32]. This is a 30-item self-rated scale to assess behavioral impulsivity.

#### Neuropsychological assessment

Subjects were evaluated with a complete neuropsychological battery based on the literature from previous research that explored cognitive functions in subjects with BPD [7–10]. The estimated IQ was evaluated with the WAIS-III vocabulary subtest. The battery consisted of several tests to evaluate three different domains: attention, memory, and executive function. In order to sum up redundant neuropsychological tests assessing these domains, three indexes were further calculated with composite score.

1. *Attention Index.* This was calculated by summing standarized scores obtained from the Symbol Digit Modality Test [33] to evaluate sustained attention and processing speed test and the inverse standarized values from Trail Making Test A [34], which requires visual exploration, numeric ordenation and viso-motor speed.
2. *Memory Index.* This was calculated by means of the standardized scores from the Buschke Selective Reminding Test [35], which explores immediate declared verbal and delayed memory.
3. *Executive index.* This index was calculated by summing standardized scores from different tests such as Controlled Oral Word Association Test (COWAT-FAS) [36] to assess verbal fluency; Trail Making Test B [34] to assess cognitive flexibility; Direct and Inverse Digit tests [37] to assess working memory; Stroop Colour-Word Interference Test [38] to evaluate inhibition control; and finally, number of categories from the Wisconsin Card Sorting Test (WCST) [39] to assess the capacity of abstraction, cognitive flexibility, elaboration of concepts and planning.

### Statistical analysis

All analyses were performed with the SPSS 19.0 software package for Windows and all hypotheses were tested with a two-sided significance level of 0.05. First, descriptive analyses were performed using chi-square test for categorical variables and *t*-test for continuous variables. Given the high number of dropouts in post-intervention and follow-up, the planned repeated measures ANOVA was substituted by the Hierarchical Linear Modeling (HLM) to investigate treatment, time and the interaction with the MIXED procedure of SPSS [40]. *Post hoc* analyses were performed with HLM to evaluate group-specific changes related to intervention. Subjects were included only if they had baseline measure and at least one post-baseline measure and all analyses were conducted on an intention-to-treat basis. The primary outcome was the change in total score of FAST but this same procedure was applied to test changes on clinical and neuropsychological variables.

## Results

### Sociodemographics and clinical characteristics

**Fig. 1:**
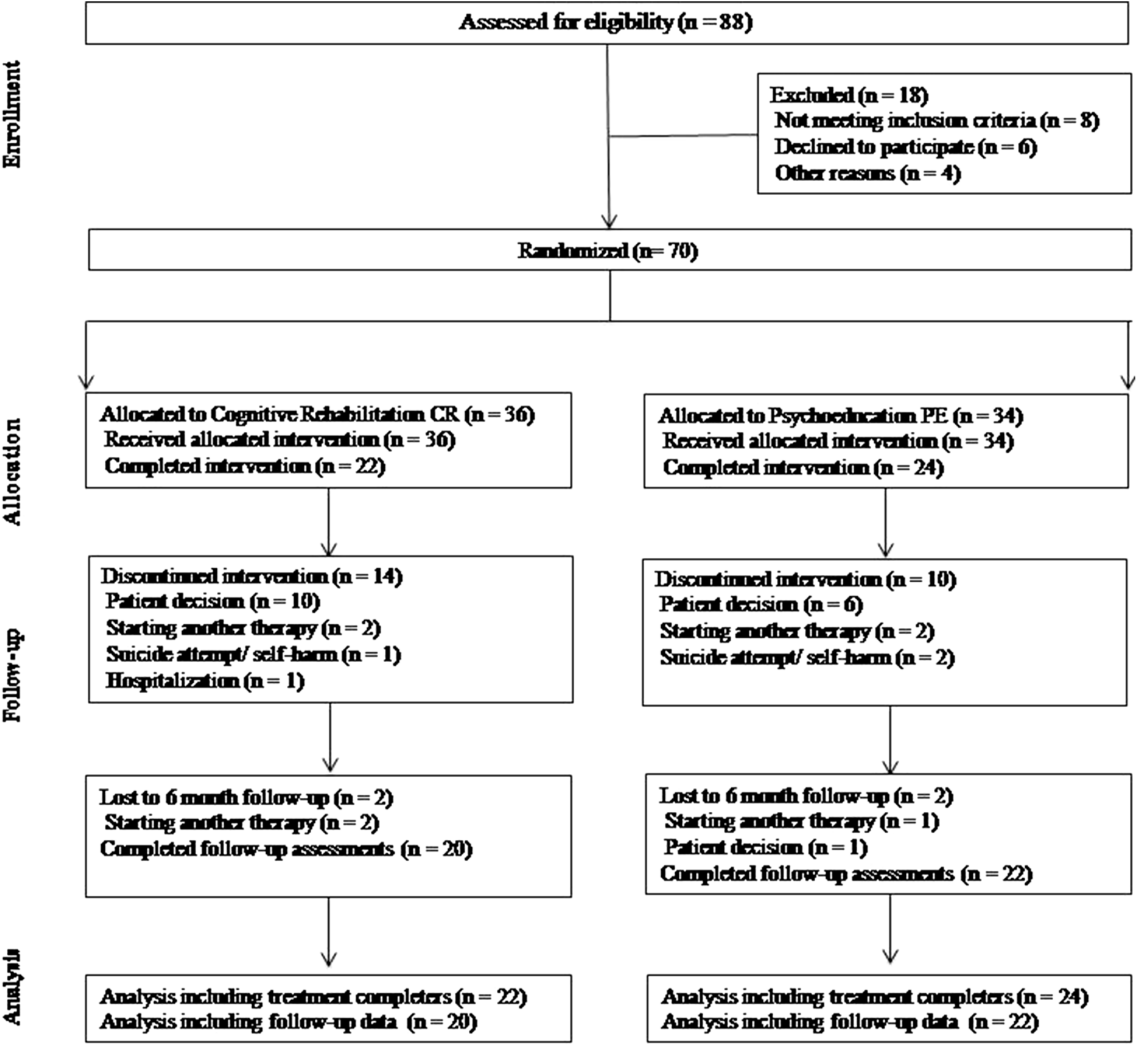
The CONSORT diagram shows the randomization of patients to cognitive rehabilitation or psychoeducation and the progress through the study

Patient flow is presented in Fig. 1. A total of 88 participants were evaluated over 20 months, 18 of whom were not included for different reasons (refusal to participate, language difficulties, no longer meeting study criteria and lost to follow-up). Finally, 70 individuals were randomized (1:1): 36 participants (51 %) to the CR arm and 34 (49 %) to the PE arm. During the intervention, 28 subjects discontinued the study without differences between groups, 16 subjects (44 %) dropped out the CR and 12 (35 %) dropped out in the case of the PE. The most reported cause for abandoning was “patient’s desire to drop out of the study” (Fig. 1). There were no significant differences at baseline between individuals that finalized the intervention and those that dropped out in terms of demographic and clinical characteristics.

As shown in Table 1, there were no significant differences between the two interventions at baseline in terms of demographic characteristics, clinical severity or pharmacological treatment. The majority of individuals were women (74 %) and more than 40 % of the sample had a long-term sick-leave. The sample had a moderate to severe clinical profile and a poor psychosocial functioning. Most participants were taking pharmacological treatment and had participated in previous psychotherapies (mainly Dialectical Behaviour Therapy) but did not have previous experience in cognitive rehabilitation or psychoeducation programme.

**Table 1:** Summary of demographics and clinical variables at baseline

	CR (*n* = 36)	PE (*n* = 34)	*P*
Gender (n/% females)	28/77.8	24/70.6	n.s.
Age	32.4 (6.04)	32.8 (8.8)	n.s.
Years of education	11.78 (4.12)	11.0 (3.33)	n.s.
Sick-leave/Unemployed (n/%)	16/44.4	13/38.2	n.s.
DIB-R	7.65 (1.35)	7.33 (1.31)	n.s.
GAF	54.74 (7.45)	56.03 (8.94)	n.s.
CGI-BPD	4.94 (0.79)	4.74 (0.93)	n.s.
Pharmacological treatment (n/%)	27 (75)	23 (67.6)	n.s.
Antidepressant	24 (66.7)	22 (64.7)	n.s.
Benzodiazepine	16 (44.4)	13 (38.2)	n.s.
Mood Stabilizer	20 (55.6)	13 (38.2)	n.s.
Antipsychotic	11 (30.6)	11 (32.4)	n.s.

Note: Values represent mean scores (SD between brackets) or otherwise specified. No significant differences between groups were observed in *χ* ^2^ test for categorical variables neither in *t*-test for quantitative ones (n.s)

Sick-leave/Unemployed: long-term sick-leave (>3 months) or for being unemployed, *DIB-R* Diagnostic Interview for Borderlines-Revised, *GAF* Global Assessment Funcioning, *CGI-BPD* Clinical Global Impression Scale for BPD

### Primary outcome

#### Psychosocial functional improvement

**Fig. 2:**
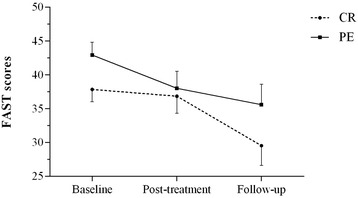
General functional improvement measured by FAST scores in CG and PE groups in pre-, post- and 6 months follow-up measurements. Note Fig. 2: Rounded and squared dots represent means and bars, standard errors. CR = Cognitive Rehabilitation; PE = Psychoeducational intervention. A time effect was observed in the HLM analysis [F(2, 41.14) = 7.54, *p* = .002]. *Post hoc* analyses showed that FAST only improved significantly in the CR condition (*p* = .018)

Table 2 presents a summary of mean scores corresponding to pre- and post-intervention as well as 6-month follow-up measurements. HLM was carried out including number of sessions as a covariable and main effects can be found in Table 2. The primary outcome of the trial (FAST) did not show main effects of group, group x time or number of sessions. There was a significant main effect of time for both treatments (baseline, post-treatment, and 6-month follow-up assessments) [F(2, 40.04) = 6.34, *p* = .004]. *Post hoc* analyses showed that CR was the intervention which showed greater improvement in the FAST (*p* = .018) (Fig. 2).

**Table 2:** Summary of pre- post-intervention and 6-months follow-up measures and significant interactions on HLM analysis

	CR				PE						
	Baseline (*n* = 36)	Post-treatment (*n* = 22)	Follow-up (*n* = 20)	*Post-hoc tests*	Baseline (*n* = 34)	Post-treatment (*n* = 24)	Follow-up (*n* = 22)	*Post-hoc tests*	HLM		
									Group	Time	Group x time
Psychosocial Functioning											
*FAST*	37.86 (1.81)	36.86 (2.55)	29.55 (2.92)	*p = .018*	42.94 (1.89)	38.01 (2.51)	35.61 (3.03)	*n.s.*	*n.s.*	*p = .004*	*n.s.*
Clinical variables											
*BSL-23*	42.32 (3.90)	42.56 (5.00)	34.91 (5.25)	*n.s.*	40.42 (4.14)	38.89 (4.86)	39.59 (5.53)	*n.s.*	*n.s.*	*n.s.*	*n.s.*
*HARS*	24.89 (1.94)	19.76 (2.62)	16.53 (2.61)	*p = .023*	21.60 (2.02)	18.61 (2.65)	11.52 (2.87)	*p = .006*	*n.s.*	*p < .001*	*n.s.*
*MADRS*	22.54 (1.60)	19.16 (1.83)	20.02 (1.98)	*n.s.*	18.67 (1.65)	16.58 (1.85)	9.91 (2.13)	*p < .001*	*p = .009*	*p < .001*	*n.s.*
*BIS*	64.53 (2.70)	65.93 (3.34)	57.14 (4.04)	*n.s.*	70.59 (2.84)	67.92 (3.33)	67.06 (4.35)	*n.s.*	*n.s.*	*n.s.*	*n.s.*
Neuropsychological variables											
*Memory Index*	–.10 (.11)	–.13 (.09)	–.15 (.11)	*n.s.*	.20 (.11)	.01 (.09)	.11 (.11)	*n.s.*	*n.s.*	*n.s.*	*n.s.*
*Attention Index*	.01 (.13)	–.07 (.15)	–.18 (.15)	*n.s.*	.10 (.14)	.29 (.15)	.35 (.15)	*n.s.*	*n.s.*	*n.s.*	*p = .020*
*Executive Index*	-.04 (.20)	.08 (.24)	–.08 (.24)	*n.s.*	-.02 (.21)	.38 (.23)	.13 (.25)	*n.s.*	*n.s.*	*n.s.*	*n.s.*

*t*-test * < .05, ** < .01, *** < .001

Note: HLM analyses correspond to pre-, post- and follow-up measures. Post-hoc were obtained by HLM per each group. *FAST* Functioning Assessment Scale Test, *BSL-23* Borderline Symptom List – 23, *HARS* Hamilton Anxiety Rating Scale, *MADRS* Montgomery-Asberg Depression Rating Scale, *BIS* Barrat Impulsivity Scale

### Secondary outcomes

#### Clinical improvement

No significant changes related to CR or PE were observed in BPD psychopathology measured by BSL-23 (*p* > .6). Regarding impulsivity measured by BIS, no significant differences were observed either (*p* = .1). Significant time effects were observed in the HLM analyses for HARS [F(2, 46.29) = 9.29, *p* < .001] and MADRS [F(2,45.63) = 8.92, *p* < .001]. An effect of group [F(1, 64.14) = 7.37, *p* = .009] and a tendency for group x time [F(2, 45.63) = 2.96, *p* = .062] was also found in MADRS scores. *Post hoc* analyses indicated significant improvements on anxiety and depression in PE group (*p* = .006 and *p* < .001, respectively) and on anxiety in the CR group (*p* = .023).

#### Neuropsychological improvement

No effect of the interventions was found for the Memory and Executive Indexes (*p* > .05). Regarding the Attention Index, a group x time interaction was found [F(2, 39.66) = 4.33, *p* = .02], although *post hoc* tests indicated no significant differences in Attention Index (*p* > .05).

## Discussion

This is the first randomized clinical trial that evaluates the efficacy and long-term effects of a cognitive rehabilitation group intervention compared with a psychoeducational group intervention in subjects with borderline personality disorder. The results show that cognitive rehabilitation exerted the greatest change on the primary outcome six months after the intervention finished but not at its end, demonstrating long-term effects of cognitive remediation. By contrast, psychoeducational intervention also showed a significant enhancement of depressive symptoms and attention functioning.

It has been suggested that cognitive remediation programs for psychiatric disorders should aim at improving general functioning beyond cognition [41]. As in a previous trial with patients suffering bipolar disorder [20], inclusion criteria of the present study required functional disability of patients in their daily activities but not necessarily an impaired cognitive profile. Therefore, this could explain the beneficial effects of cognitive rehabilitation on psychosocial functioning without observing neuropsychological changes, neither at the end of the intervention nor in the follow-up assessment. Strikingly, our results showed that some cognitive deficits, as measured with neuropsychological tests, remained unchanged after cognitive rehabilitation although patients exhibited fewer difficulties and better functioning after intervention, regardless of whether it was cognitive rehabilitation or psychoeducation. This was unexpected, as the hypothesis was that enhancement of cognitive functioning should have accounted for improvements in functional outcomes, at least in part. Two explanations are possible: the deficits of these patients were too mild to be improved with the programs or there may have been a type II error, *i.e.,* the sample was not big enough to detect differences in neuropsychological data. Alternatively, and considering recent evidences [16, 17], it is also possible that the neuropsychological performance of subjects with BPD would be affected by emotional distress. Further studies that evaluate intervention effects on the cognitive function in BPD should control for this variable. In any case, cognitive rehabilitation emphasized the use of compensatory skills to cope with daily life difficulties. Psychoeducational intervention would have exerted similar effects as patients acquired deeper insight into their illness and new strategies to cope with stress.

Amongst psychiatric disorders, neurocognitive rehabilitation has been efficiently tested for schizophrenia with the Integrated Psychological Therapy or IPT [42] and the Cognitive Remediation Therapy or CRT [18]. More recently, it has been tested for affective and bipolar disorders with significant efficacy [19, 20]. To date, there are no consistent data on neuropsychological remediation for subjects with BPD and only preliminary and uncontrolled communications of positive experiences have been published [21, 22]. No significant differences between CR and PE were found, but both interventions improved general functioning, which provides evidence of the need for therapeutic strategies focused on psychosocial difficulties of psychiatric patients. The lack of an inactive arm such as treatment as usual (TAU) might have prevented the observation of more robust differences.

Our results suggest that the general functional improvement observed in BPD is independent of clinical and neuropsychological changes. Long-term follow-up studies also show that clinical and general functioning improvements are not always related because high rates of clinical remission are not always associated with better psychosocial functioning [3, 4]. These results are in concordance with a recent study in bipolar disorder where a delayed improvement on general functioning was also found without clinical or neuropsychological changes [20], and are strengthened even more by the fact that conditions of the trial were controlled until the end of the follow-up. What is clear is that psychosocial functioning needs to be specifically addressed to obtain a global recovery of BPD patients.

Psychoeducation impacted on clinical depressive and anxiety symptoms as well as on attention domain compared with CR. Our PE was partially based on “Awareness of Borderline Personality Disorder”, the first step of STEPPS programme that had previously been demonstrated to improve clinical symptoms in BPD [27]. A previous study comparing psychoeducational training with waiting list also showed efficacy in symptoms such as impulsivity or unstable relationships but no improvement in psychosocial functioning [43]. It is worth mentioning that PE efficacy for clinical and neuropsychological enhancement was observed with half of the sessions of CR intervention.

The following limitations have to be taken into consideration. The final sample size was limited due to the high number of dropouts. Therefore, the present findings need replication in larger samples. Such small numbers did not allow us to perform analyses by domains of the FAST, as done in a previous study [20]. Given that this was a multicenter trial, the representativeness of the sample with regard to subjects with BPD from Spain can be acknowledged. Individual motivation to change and groupal procedure are key factors for some types of psychotherapeutic interventions. These factors were not evaluated in this study, and could have affected the findings, particularly for CR, which normally requires additional efforts and a more personalized intervention. In any case, CR was compared with an active intervention although with lower duration and intensity, and not a weaker treatment condition such as treatment as usual or waiting list. The presence of a third arm with an inactive treatment would probably have been useful to confirm a significant efficacy of both interventions. The influence of pharmacological effect was not evaluated, but most patients were treated without differences between treatment arms. Finally, we did not control other non-specific factors that could affect psychological functioning as social relationships, family support, regular social activity stimulated by trainings, etc.

## Conclusions

In summary, preliminary results from the present study seem to suggest that both cognitive rehabilitation and psychoeducational interventions could be efficacious in subjects with BPD to treat functional disabilities in daily life. These interventions can be easily implemented in mental health settings and provide advantages to improve general functioning, which frequently remains affected after clinical remission in long-term follow-up [2–4]. The present results highlight the need for further research in order to better determine the specific impact of these interventions on psychosocial functioning in BPD. Specifically, investigating the combined effect of cognitive rehabilitation with other BPD-specific psychotherapeutic models (i.e., DBT or Mentalization-Based Therapy) would be of special interest. Doing so may provide useful information to better determine if adding cognitive rehabilitation would amplify the efficacy of well-established BPD treatments.

## Additional file

Additional file 1: Figure S1. CONSORT checklist. (PDF 684 kb)
